# The efficacy of a symptom management platform among gastric cancer survivors: a study protocol for a randomized controlled trial

**DOI:** 10.1097/SP9.0000000000000047

**Published:** 2025-05-27

**Authors:** Jeong Ho Song, Joongyub Lee, Jae Seok Min, Hyungkook Yang, Jong Hyuk Yun, Hayemin Lee, Eun Young Kim, Su Mi Kim, Sol Lee, Ki Bum Park, Geum Jong Song, Ji Ho Park, Bang Wool Eom, Hoon Hur, Sang Ho Jeong

**Affiliations:** a Department of Surgery, Ajou University School of Medicine, Suwon, Republic of Korea; b Department of Preventive Medicine, Seoul National University College of Medicine, Seoul, Republic of Korea; c Department of Surgery, Korea University College of Medicine, and Division of Foregut Surgery, Korea University Anam Hospital, Seoul, Republic of Korea; d Lunit CARE Inc, Seoul, Republic of Korea; e Department of Surgery, Soonchunhyang University Cheonan Hospital, Cheonan, Republic of Korea; f Division of Gastrointestinal Surgery, Department of Surgery, Bucheon St. Mary’s Hospital, College of Medicine, The Catholic University of Korea, Bucheon-si, Republic of Korea; g Department of Surgery, Uijeongbu St. Mary Hospital, College of Medicine, The Catholic University of Korea, Uijeongb, u-si, Republic of Korea; h Department of Surgery, CHA Bundang Medical Center, CHA University, Seongnam, Republic of Korea; i Department of Surgery, Seoul Medical Center, Seoul, Republic of Korea; j Department of Surgery, Kyungpook National University Chilgok Hospital, Daegu, Republic of Korea; k Department of Surgery, Gyeongsang National University School of Medicine & Gyoengsang National University Changwon Hospital, Changwon, Republic of Korea; l Center for Gastric Cancer, National Cancer Center, Goyang, Republic of Korea

**Keywords:** digital platform, gastrectomy, gastric neoplasm, quality of life, survivors

## Abstract

**Background:**

Patients who undergo gastrectomy for gastric cancer experience gastrointestinal symptoms, psychological responses, and social problems. These factors reduce a patient’s quality of life (QoL) after surgery. A web-based platform (Wecare^®^) has been developed to address distress and provide solutions. This study aimed to evaluate whether Wecare® improved the QoL of patients who underwent gastrectomy.

**Methods:**

A total of 88 patients who undergo gastrectomy for gastric cancer will be randomly allocated to either the “Wecare^®^” group or the control group at a 1:1 ratio. After using the “Wecare^®^” platform for the first 22 patients and making modifications, the next 66 patients will be randomized equally. The primary outcome of this trial is the QoL among gastric cancer survivors (KOQUSS-40). The values of weight change, nutritional index change, KOQUSS-40 questionnaire compliance, self-efficacy, physical activity change, and satisfaction will be compared between the two groups as secondary outcomes. The investigator will follow-up with the patients at 1, 3, and 6 months after surgery at the outpatient clinic.

**Discussion:**

This is the first randomized controlled trial to analyze the usefulness of a symptom management platform (Wecare^®^) among gastric cancer survivors. This study aimed to verify the efficacy of web-based platforms in relieving discomfort after gastric cancer surgery. Future large-scale clinical trials are planned.

**Highlights**

- First randomized controlled trial evaluating a digital symptom management platform (Wecare®) for gastric cancer survivors after gastrectomy.
- Wecare® provides personalized, algorithm-driven education and expert feedback based on the KOQUSS-40 questionnaire.
- The platform enables continuous symptom monitoring and enhances postoperative quality of life.
- This study supports the feasibility of integrating digital health tools into survivorship care after cancer surgery.
- Findings may lay the groundwork for scalable, patient-centered digital platforms in oncology.

## Introduction

### Background and rationale {6a}

Surgery remains the mainstay of gastric cancer treatment. With the increasing prevalence of early gastric cancer and improved survival rates, especially in East Asia due to nationwide screening programs, the number of long-term survivors has grown steadily^[1–3]^. Consequently, there is a rising focus on improving quality of life (QoL) after gastrectomy. Patients often experience a range of physical symptoms known as post-gastrectomy syndrome, along with psychological distress and social challenges that significantly affect their daily lives^[4,5]^. To address these unmet needs, digital health solutions have gained attention^[6–10]^. Previous randomized clinical trials have examined the effect of smartphone-based interventions in patients with cancer, and the results have revealed that these interventions enhance recovery quality and provide comparable satisfaction with care to conventional in-person follow-ups^[11–15]^.

We care® is a novel, web-based platform specifically designed to support gastric cancer survivors after gastrectomy. Developed by the KOQUSS group, it integrates an intelligent screening system based on the validated KOQUSS-40 questionnaire to identify patient-specific symptoms and needs. Based on this assessment, the platform provides personalized educational content and expert-driven recommendations tailored to the patient’s recovery phase. Furthermore, the results are shared with physicians to facilitate more informed and efficient consultations during outpatient visits.

To the best of our knowledge, this is the first randomized controlled trial to evaluate the impact of a digital symptom management platform on QoL after gastric cancer surgery. This study aims to assess whether Wecare® can enhance postoperative recovery and support long-term survivorship in a cost-effective, scalable way.

### Objectives {7}

The Korean Quality of Life in Stomach Cancer Patients Study Group (KOQUSS) in the Korean Gastric Cancer Association developed a survey tool (KOQUSS-40, Table 1) to appropriately assess the QoL of gastric cancer patients who have undergone gastrectomy and developed a digital platform (Wecare^®^) based on the KOQUSS-40^[16]^. The Wecare^®^ platform conducts assessments by screening for patient needs and symptoms based on questionnaires. It provides patients with reporting and relevant information (including content and videos). The assessment results were also shared with physicians for use as patient information during outpatient clinic visits. **In this study, we propose a randomized controlled trial to compare QoL after gastrectomy in patients with and without Wecare^®^ support.**

**Table 1:** KOQUSS questionnaire*

Over the past week	Very satisfied	Slightly satisfied	Slightly dissatisfied	Very dissatisfied	
1. How is your overall health?	□	□	□	□
2. How is your overall quality of life?	□	□	□	□
3. Are you satisfied with your cancer treatment?	□	□	□	□
4. Are you satisfied with the surgical scars?	□	□	□	□
5. Are you satisfied with the cost of your cancer treatment?	□	□	□	□

Over the past week	Not at all	Somewhat	Quite	Very much
6. Do you feel uncomfortable because you eat slowly in social situation?	□	□	□	□
7. Do you feel uncomfortable because you eat too often?	□	□	□	□
8. Do you feel uncomfortable carrying on with daily life due to lack of energy?	□	□	□	□
9. Has the amount of food intake decreased compared to before surgery?	□	□	□	□
10. Has your appetite reduced?	□	□	□	□
11. Do you feel full even if you ate small amount of food?	□	□	□	□
12. Do you feel food gets stuck in the throat when eating?	□	□	□	□
13. Do you feel something is stuck in the throat when drinking water?	□	□	□	□
14. Do you feel discomfort with fullness in your upper abdomen after eating?	□	□	□	□
15. Do you feel food is regurgitating back up (coming up)?	□	□	□	□

Over the past week	Not at all	Somewhat	Quite	Very much
16. Do you get bitter water from the stomach to your mouth?	□	□	□	□
17. Do you have any burning sensation in your chest?	□	□	□	□
18. Have you ever had abdominal pain with bloating after a meal?	□	□	□	□
19. Have you ever had a heart palpitation after a meal?	□	□	□	□
20. Have you ever had a blush or hot face after a meal?	□	□	□	□
21. Have you ever turned pale after a meal?	□	□	□	□
22. Have you ever had a sudden cold sweat before?	□	□	□	□
23. Have you ever had diarrhea after a meal?	□	□	□	□
24. Have you ever had any abdominal pain that is not related to eating?	□	□	□	□
25. Do you feel uncomfortable due to gas pains?	□	□	□	□
26. Do you feel uncomfortable due to frequent bowel movement?	□	□	□	□
27. Do you feel uncomfortable due to frequent wind?	□	□	□	□
28. Do you feel uncomfortable with constipation?	□	□	□	□
29. Do you have hard stool?	□	□	□	□
30. Have you ever been nervous?	□	□	□	□
31. Have you ever been depressed?	□	□	□	□
32. Have you ever felt lethargic?	□	□	□	□
33. Do you have insomnia?	□	□	□	□
34. Have you ever suddenly been dizzy and wanted to sit down?	□	□	□	□
35. Are you worried about losing weight?	□	□	□	□
36. Are you reluctant to go to the bathroom or pool because of a surgical scar?	□	□	□	□
37. Are you worried about your stomach cancer coming back?	□	□	□	□
38. Do you have pain in your surgical scar?	□	□	□	□
39. Do you have itchiness in your surgical scar?	□	□	□	□
40. Do you have financial difficulty because of the cost of your cancer treatment?	□	□	□	□

* Questions below ask you about your post-surgery health, quality of life, and satisfaction. Please check (v) to indicate the extent to which you are satisfied in regard to how you have been past week.

### Trial design {8}

**Figure 1.**
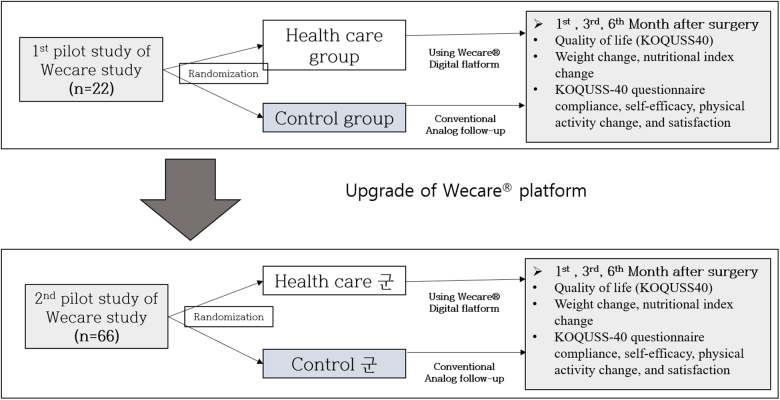
Flow diagram of the Wecare^®^ study.

This randomized controlled multicenter trial with two parallel groups aims to evaluate the efficacy and safety of a web-based intervention program, named “Wecare^®^.” The primary endpoint will be QoL among survivors of gastric cancer. The investigator will examine the patients 1, 3, and 6 months after surgery (Fig. 1).

## Methods: participants, interventions, and outcomes

### Study setting {9}

This study enrolled participants who underwent radical gastrectomy at 11 participating teaching hospitals in the Republic of Korea, one of the countries with the highest incidence of gastric cancer. This study was approved by the Institutional Review Board of the Hospital.

### Eligibility criteria {10}

Patients with gastric cancer aged 19–75 years who are indicated for gastrectomy will be eligible for this study. To be included, the patient should be operable and gastric cancer should be surgically removable (stages I–III). Patients who could not be followed up regularly, were illiterate, or did not consent to participate in the study were excluded. In addition, patients with severe psychiatric disorders, significant comorbidities, inability to communicate effectively, or other medical or personal conditions deemed inappropriate for participation by their attending surgeon were also excluded. Informed consent was obtained from all participants.

### Who will obtain patient informed consent? {26a}

Investigators will thoroughly explain the trial details to the potential participants and provide patient informed consent. Participants were given sufficient time to consider their decision to join the trial. They could then sign the informed consent form and were free to withdraw at any time during the trial. Written informed consent was obtained from the patient for publication. A copy of the written consent is available for review by the Editor-in-Chief of this journal on request.

### Additional consent provisions for the collection and use of participant data and biological specimens {26b}

No additional biological samples were collected for this study. However, standard clinical laboratory data, including hemoglobin, albumin, and lymphocyte counts, were obtained as part of routine postoperative care and used for evaluating nutritional status.

## Interventions

### Explanation for the choice of comparators {6b}

**Figure 2.**
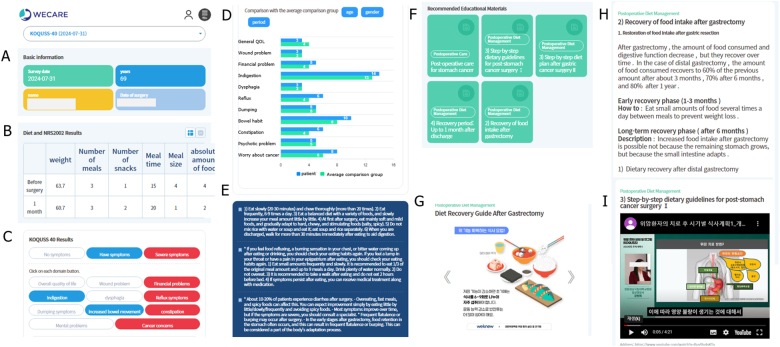
Screenshots from Wecare^®^ application used by patients. **A** shows the patient’s basic information. **B** displays the diet and NRS2002 results. **C** evaluates the scores for each domain of the KOQUSS-40 questionnaire, where white indicates no to mild symptoms, blue represents intermediate symptoms, and red shows severe symptoms. **D** compares the patient’s values in each domain with the average values of a small group matched for age, gender, and operation period within an existing patient group. **E** provides automated responses regarding education and treatment for domains showing severe symptoms. **F** contains recommended educational materials from medical professionals about symptoms based on the patient’s postoperative timeline. **G** features educational materials in a card news format, primarily composed of images. **H** provides educational materials centered on text. **I** consists of educational materials presented in video format.

In the intervention group, the participants were asked to use the Wecare^®^ platform (Fig. 2A~I). The Wecare^®^ platform administers QoL questionnaires, including the KOQUSS-40, following gastrectomy for gastric cancer. Based on participants’ responses, the platform automatically generates expert recommendations from gastric cancer specialists based on participants’ responses according to the timing of surgery after gastrectomy (Fig. 2C~E). Furthermore, the Wecare^®^ platform offers educational materials for gastric cancer to address the participants’ questions about the disease (Fig. 2F~I). Participants will be free to use the platform but will be asked to visit the Wecare^®^ platform at least once a month and answer questionnaires. The participants will visit an outpatient clinic before surgery and at 1, 3, and 6 months after surgery.

In the control group, participants will be asked to complete written questionnaires in the outpatient clinic before surgery and 1, 3, and 6 months after surgery. Based on the questionnaires, the clinician provides a solution at an outpatient clinic. However, the Wecare^®^ platform is not available.

### Intervention description {11a}

To understand the feasibility of participant recruitment or study design at all participating sites, we planned to determine the number of participants in two phases. In the first phase, 22 patients will be recruited from 11 institutions (two patients per institution) to collect patient data using the draft protocol and correct any problems, and 66 patients will be recruited from 11 institutions (6 patients per institution) to measure outcome variables preoperatively and at 1, 3, and 6 months postoperatively, according to the revised protocol. The number of subjects required for the main study was calculated based on the data from the second phase. Each participating hospital is expected to enroll a sufficient number of eligible patients because the incidence of gastric cancer in South Korea is very high and participating institutions perform a large number of gastric cancer surgeries each year.

The Wecare® platform aims to improve postoperative quality of life through multiple integrated mechanisms. Patient-reported outcomes are collected via the KOQUSS-40 questionnaire at regular intervals, enabling continuous monitoring of physical, psychological, and social symptoms. Based on these responses, the platform delivers algorithm-based personalized content, including tailored educational resources (text, image, and video formats) and automated feedback aligned with each patient’s symptom profile and recovery phase. The platform stratifies symptoms by severity and provides immediate guidance or recommendations for self-management. These results are also shared with physicians before outpatient visits, facilitating more informed clinical decision-making. This integrated, patient-centered digital approach is expected to promote self-efficacy, reduce symptom burden, and ultimately enhance overall QoL following gastrectomy.

### Criteria for discontinuing or modifying allocated interventions {11b}

There were no predetermined criteria for discontinuing or modifying the interventions assigned to participants. All individuals participated voluntarily and had the option to discontinue the study at any time for any reason without negative consequences.

### Strategies to improve adherence to interventions {11c}

This clinical trial will recruit patients with cancer who will require regular follow-up to assess cancer recurrence. We anticipate that this will improve the adherence to the study protocol.

### Relevant concomitant care permitted or prohibited during the trial {11d}

This clinical trial is not related to the surgery or drug itself but rather to the quality of daily life after surgery. Therefore, no relevant concomitant postoperative care or intervention was permitted or prohibited during the trial.

### Provisions for posttrial care {30}

There will be no provision for patients who participated in the trial. Posttrial care followed the usual standards of care after gastric cancer surgery.

### Outcomes {12}

The primary outcome of this trial was the QoL of the gastric cancer survivors (KOQUSS-40). The KOQUSS-40 questionnaire was developed and validated to evaluate the QoL of patients with gastric cancer who underwent gastrectomy. The questionnaire included 40 items across 11 domains, focusing on QoL related to postgastrectomy symptoms^[16]^. Using this questionnaire, we will compare each item and domain score between the groups before surgery and at 1, 3, and 6 months after surgery in the outpatient clinic.

Weight, nutritional parameters, questionnaire compliance, self-efficacy, and physical activity were compared between groups as secondary outcomes. Nutritional parameters, such as hemoglobin, albumin, and lymphocytes, and the prognostic nutritional index will be measured using common laboratory tests. Information on satisfaction with the Wecare^®^ program will also be collected.

### Participant timeline {13}

**Figure 3.**
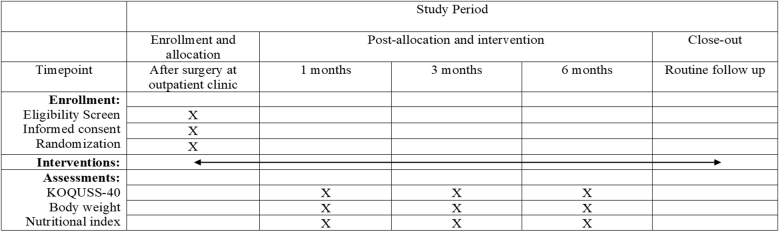
Timeline of the study period.

The participant timeline is shown in Figure 3.

### Sample size {14}

This pilot study primarily aimed to evaluate the feasibility and preliminary efficacy of the intervention; hence, formal sample size calculation based on statistical power was not performed^[17]^. However, the sample size of 88 participants was determined based on prior feasibility studies and expected effect sizes derived from previous QoL-related interventions. The initial cohort of 22 patients was used to optimize the protocol, followed by a randomized allocation of the remaining 66 participants.

### Recruitment {15}

Recruitment will be conducted through announcements in the outpatient departments of each participating institution. Investigators will contact patients who wish to participate in this clinical trial and determine participation based on the inclusion and exclusion criteria.

## Assignment of interventions: allocation

### Sequence generation {16a}

Eligible patients will be randomly assigned to either the control or experimental group in a 1:1 ratio. Medical statisticians will perform stratified randomization. This randomization will be performed based on an independent computer-based sequence generated from the implementation of the dynamic algorithm as the stratifying variable.

### Concealment mechanism {16b}

Randomization will be performed as block randomization with a 1:1 allocation. To achieve randomization, a computer-generated sequence methodology will be used, which will ensure that both the randomization methodology and the allocation sequence are concealed from both the investigator and participants.

### Implementation {16c}

Randomization will be performed by an independent medical statistician, who will generate a stratified randomization list for the allocation sequence. Investigators will enroll and assign participants to the interventions.

## Assignment of interventions: blinding

### Who will be blinded {17a}

Clinician and patient blinding is impossible because of the nature of the trial regarding whether or not to apply the Wecare^®^ platform. However, the outcome data will be analyzed by statisticians blinded to group allocation to reduce assessment bias.

### Procedure for unblinding if needed {17b}

Because the investigators and participants will be aware of the allocation group, unblinding will not be required.

## Data collection and management

### Plans for assessment and collection of outcomes {18a}

The baseline demographic, surgical, and pathological characteristics of the patients and outcome variables were collected. Demographic and clinical characteristics were collected using an electronic case report form based on the participants’ electronic medical records. In the intervention group, questionnaire-based data will be collected using tablets at every planned visit, where the electronic questionnaire will be designed to warn the respondent about any missing values or outliers. A written questionnaire was administered to the control group. Because the data will be stored in the data center of the KOCAS ecosystem, central edit checking will be performed for all input data. A manual review of patient data will be performed in case of any error from the edit check.

### Plans to promote participant retention and complete follow-up {18b}

The participants will include patients with gastric cancer who need regular follow-up to monitor for cancer recurrence. We anticipate that participants will attend regular follow-ups because of concerns about cancer recurrence.

### Data management {19}

The study data will be collected and recorded electronically and stored securely on computers and hard drives at each institution. A data management system was established so that data can be accessed from the center that enters the data and the central data center. This ensured that the data were ready for accuracy checks and validation throughout the study. All data will be quality-assured, and data locking will precede the analysis.

### Confidentiality {27}

The datasets will be anonymous, and the manuscript will only include aggregate data that do not reveal the identities of the individual participants.

### Plans for collection, laboratory evaluation, and storage of biological specimens for genetic or molecular analysis in this trial/future use {33}

Not applicable, as no biological specimens were collected in this study.

## Statistical methods

### Statistical methods for primary and secondary outcomes {20a}

Intention-to-treat (ITT) analysis, which includes all patients who are randomly assigned, will be used for primary and secondary outcomes. ITT analyses provide a conservative estimate of the treatment effect and identify statistically significant differences. If consent withdrawal or dropout occurs during follow-up, the data up to the time of consent withdrawal or dropout will be used. Comparative analyses of dropout rates by arm will also be conducted. Adherence to the Wecare^®^ program will be assessed under two conditions: Wecare^®^ webpage visits and completion of the KOQUSS-40 questionnaire on the Wecare^®^ webpage. Participants who visited the webpage at least once a month during the study period and completed all questionnaires on the webpage at 1, 3, and 6 months after surgery were considered to have high adherence and were included in the per-protocol analysis. A per-protocol analysis will also be performed to evaluate the treatment effect with high compliance.

The demographic data and baseline characteristics of all randomized participants will be presented as means and standard deviations for continuous variables and as frequencies and percentages for categorical variables. A summary of the primary and secondary outcomes will also be provided, which will be compared at each time point using the chi-squared test, Fisher’s exact test, Student’s t-test (two samples), or Mann–Whitney U test, as appropriate. The primary outcome variable (QoL) will be assessed using a linear mixed model to account for the clustering of four measurements in a single patient to evaluate the effect of the Wecare^®^ intervention. For the secondary outcome variable, we present the results of between-group comparisons, considering the same clustering.

As the measurement of outcomes will be repeated for four visits, there may be some missing values for the outcome variables. For KOQUSS-40, if more than or equal to half of the items from a scale are answered, all the completed items can be used by applying the standard equations for calculating the scale scores. However, if less than half of the items are answered, the scale score is considered missing. Missing scores were addressed using the multiple imputation method. All statistical tests will be 2-sided, and a *P*-value > 0.05 will be considered to indicate statistical significance. The data will be analyzed using R (version 4.2.1; R: a language and environment for statistical computing, R Foundation for Statistical Computing, Vienna, Austria).

### Interim analyses {21b}

We do not have any plans to conduct interim analyses because this trial does not cause harm to patients. However, the platform could be modified after analyzing the results of the first 22 patients.

### Methods for additional analyses (e.g., Subgroup analyses) {20b}

Subgroup analyses will be performed according to the type of gastrectomy. Additionally, to explore differential effects among patient subsets in terms of age, baseline health status, and comorbidities, we will conduct subgroup analyses.

### Methods in analysis to handle protocol nonadherence and any statistical methods to handle missing data {20c}

The loss to follow-up will be minimized because of the characteristics of patients with cancer. If more than 5% of the data for any variable were missing, multiple imputations were used to handle the missing values.

### Plans to give access to the full protocol, participant-level data, and statistical code {31c}

The full protocol and final datasets are available from the corresponding author upon reasonable request.

## Oversight and monitoring

### Composition of the coordinating center and trial steering committee {5d}

The authors will coordinate and steer this study.

### Composition of the data monitoring committee and its role and reporting structure {21a}

We do not have a data monitoring committee.

### Adverse event reporting and harms {22}

This clinical trial itself did not harm the participants. However, if patients who need immediate treatment are screened through the Wecare^®^ platform, they are asked to visit the hospital for evaluation.

### Frequency and plans for auditing trial conduct {23}

The study data will be maintained in accordance with the Good Clinical Practice requirements of investigators. The original study data and information will be securely stored for at least 3 years after the completion of the study. Data monitoring reports will be submitted to the ethics committee every 3 months.

### Plans for communicating important protocol amendments to relevant parties (e.g., trial participants and ethical committees) {25}

If the protocol requires modification, it will be reviewed again by an ethics committee. Upon approval, the trial registry and protocol will be updated.

### Dissemination plans {31a}

These findings will be published in peer-reviewed journals and disseminated through scientific and academic conferences.

## Discussion

This randomized controlled trial aimed to analyze the efficacy of a symptom management platform (Wecare^®^) among gastric cancer survivors across nine institutions in the Republic of Korea. The primary objective of this study was to determine the QoL (KOQUSS-40) of gastric cancer survivors after gastrectomy. To the best of our knowledge, this is the first study to verify the efficacy of web-based platforms in relieving discomfort after gastric cancer surgery.

Patients who have undergone gastrectomy for gastric cancer experience changes in their daily lives. Several tools have been introduced to assess QoL in gastric cancer patients, such as the European Organization for Research and Treatment of Cancer Quality of Life Questionnaire (EORTC QLQ-STO22), Functional Assessment of Cancer Therapy-gastric (FACT-Ga), and Post Gastrectomy Syndrome Assessment Scale (PGSAS-45)^[18,19]^. Due to limitations in being unable to comprehensively encompass or statistically verify patients’ gastrointestinal symptoms, the KOQUSS developed and validated the KOQUSS-40 questionnaire^[16]^. The symptom management platform (Wecare^®^) not only provides information about stomach cancer but also checks the patient’s discomfort through the KOQUSS-40 questionnaire and automatically provides a solution according to the patient’s symptoms based on the answers to the questionnaire. Wecare® is expected to not only address patients experiencing discomfort after surgery but also help reduce medical costs by eliminating unnecessary outpatient visits.

If the Wecare® platform demonstrates clinical efficacy, it could represent a paradigm shift in postoperative care for gastric cancer survivors. Traditionally, symptom monitoring and patient support after gastrectomy have heavily relied on face-to-face outpatient visits, which are often infrequent and reactive in nature. In contrast, Wecare® enables continuous, proactive symptom monitoring and timely feedback through a web-based system, empowering patients to engage in their own recovery process. The integration of personalized, algorithm-driven recommendations based on validated QoL data may not only improve patient outcomes – such as nutritional status, physical activity, and psychological well-being – but also reduce unnecessary clinic visits and optimize health care resource utilization. Therefore, this study has the potential to lay the groundwork for a scalable, patient-centered digital model in survivorship care after cancer surgery.

Recent studies have highlighted the expanding role of artificial intelligence and digital platforms in clinical oncology. For instance, comparative informatics analyses have demonstrated how machine learning approaches can be used to evaluate treatment strategies such as neoadjuvant versus adjuvant immunotherapy, providing new insights into personalized cancer care strategies^[20]^. Furthermore, the application of advanced AI models such as AlphaFold has accelerated progress in molecular biology and drug discovery, underscoring the transformative impact of machine learning in biomedical research^[21]^. In line with these advancements, the Wecare® platform represents a novel application of intelligent, patient-centered digital technology in the field of surgical oncology. By integrating validated QoL assessments with automated screening and personalized feedback, Wecare® has the potential to serve not only as a supportive tool for symptom management but also as a foundation for future AI-assisted clinical decision-making systems. This trial thus provides important reference value for the development of scalable, data-driven platforms that enhance long-term survivorship care in cancer patients.

In this clinical trial, we aimed to verify whether the QoL of gastric cancer patients improved by providing the Wecare^®^ platform to identify discomfort after gastrectomy and provide immediate feedback.

This study also has certain limitations. Typically, symptoms tend to stabilize approximately one year after gastric cancer surgery; however, the follow-up period in this study was limited to only six months postoperatively. This limitation is due to the time required for the development of the WeCare program and the duration constraints imposed by government-funded research. In future large-scale clinical studies, it will be necessary to extend the follow-up period to at least one year to address this issue appropriately.

## Conclusions

This is the first multicenter, prospective, randomized controlled trial to validate the efficacy of the digital platform Wecare^®^ in patients with gastric cancer who underwent gastrectomy. We expect that gastric cancer patients who use the Wecare^®^ platform will have a better QoL than those who do not. Recently, gastric cancer has been diagnosed at an early stage, especially in East Asia, and as medical science has developed, the number of gastric cancer survivors is increasing. Considering the cost-effectiveness of infrequent outpatient treatment and the ability to improve QoL without consulting a doctor in person, the web-based digital platform Wecare^®^ will be a useful treatment tool for gastric cancer survivors who undergo gastrectomy.

## Data Availability

The datasets used in the current study are available from the corresponding author upon reasonable request.

## Research registration unique identifying number (UIN)

This trial was registered at ClinicalTrials.gov (NCT06395935) on 1 April 2024. The first patient was enrolled after completing an individual institutional IRB.
